# Address of Dr. T. D. Wooten, President Board of Regents. Delivered Before the Texas State Medical Association, by Invitation, on the Status of the State University

**Published:** 1887-06

**Authors:** Thos. D. Wooten

**Affiliations:** President Board of Regents


### THE UNIVERSITY OF TEXAS.

By THOS. D. WOOTEN, M. D., President Board of Regents.

*Read by Invitation before the Convention of Texas State Medical Association, Nineteenth Annual Meeting, at Austin, Texas, April, 1887.*

[Contributed to Daniel's Texas Medical Journal.]

IT is most natural and proper that this, the State Medical Association, should inquire into and desire to be informed as to the present status and future prospects of the State University of Texas.

As President of the Board of Regents of that institution, I deem it my duty and privilege to impart to your body whatever information I may, and to make such comments and offer such suggestions as are prompted and warranted by the existing state of facts. What I shall say should not in any sense be considered as an official utterance of the Board, but simply as the result of my own observations and experience while a member thereof, and as entitled to some consideration because of my necessary familiarity with the condition and needs of the institution.

The interest and identity of motive of the medical profession of Texas, in and with its State University, are not merely and solely on account of the desire to see the meaical department of the institution inaugurated and in successful operation, but are and should be based on broader and more catholic considerations. As men cherishing a respect for science and a veneration for the early efforts of those who baptised its future pursuit and perfection with their blood, and consecrated its accomplishment with the first acts of their valor and wisdom—as doctors and as citizens of Texas—we are one and all deeply concerned in the speedy and successful establishment of the University in all its contemplated branches. If there ever has been among men a class or profession which has realized the necessities of mankind, made sacrifices for humanity, sympathized with its weaknesses and sought to remedy its defects, and, withal, contributed to the advancement of civilization and science, it has certainly been the medical profession. Therefore, from us, above all others, it should be confidently and justly expected that we will cordially and earnestly support and foster an institution whose success and growth are so thoroughly identified, not only with the culture and perfection of our own peculiar science, but with the general and material prosperity of our State, and with the advancement of that higher learning which makes the whole world kin. In this great enterprise we cannot afford to be quiescent or indifferent. It invites our energies as men desirous of securing to our race the highest boons of enlightment and intellectual attainments. It appeals to us as an undertaking involving the elevation of our own profession, and thereby the increased happiness of mankind.

Recall, if you please, the objects and impressions of the men who conceived and provided for this University. They intended it to be the cap-stone of a completed pyramid of thorough public instruction; they meant it for the crowning glory of a great system of education, wherein every son and daughter of Texas, without price, should be entitled to receive the fullest benefits of all that might be known or discovered in the realms of science and philosophy, and come forth fully equipped to pursue and to prosecute with success any and every branch of practical learning or speculative research. This was the magnitude of their ambition—this the ample scope of their plan for the future education of the children of Texas.

Nor did they fail to supply the means for carrying into practical effect this magnificent conception.

The declaration of Texan independence, in 1836, announced the fundamental idea of the men who founded the Republic, in bold and broad assertion, that "it is an axiom of political science that unless a people are educated and enlightened, it is idle to expect the continuance of civil liberty or the capacity for self-government;" and the Constitution of the Republic demanded the earliest possible establishment of a general system of public education. Subsequently, the Congress, by the act of January 14, 1839, appointed commissioners to locate the capital. Austin was selected, and forty acres of the most beautiful and elevated portion of the new city were especially dedicated and set apart for the site of the future State University. It is on that ground that the present incomplete but imposing University edifice is now located, and the purpose cherished by the men who chose that location should not be defeated by any sort of parsimony, or by any line of policy, which will prevent that beautiful site from being crowned by a finished temple of free and higher learning.

Again, on January 29, 1839, fifty (50) leagues of land were especially set aside for University education and ordered to be surveyed and located, which was accordingly done. These lands were located in what are now the most populous and wealthy counties of the State. Most of them have been sold and the proceeds invested in Texas State bonds. A small portion remains, and there are certain lands in McLennan county belonging to the University, under this act, which are now in litigation.

In 1858, under the administration of Governor E. M. Pease, an act was passed to establish the University and to provide for its successful maintenance and support. The preamble to that act expresses most forcibly the high aims sought to be attained, and the necessity for early and efficient support of the institution forecast by the fathers of the Republic.

Read in the light of subsequent events and later policies, the act of 1858, like many other acts of liberal legislation inaugurated at that time, is a melancholy commentary on the degenerate and narrow conceptions of more modern law makers. In fact, it is a remarkable truth, and one not encouraging to the symptoms of patriotism and civilization of these latter days, that from 1836 to 1858 the continuous and consistent policy of both the Republic and State of Texas was one of enlightened, liberal, generous and munificent devotion to the advancement and proper endowment of the University, as the means and the measure of our highest culture and civilization; while from 1859 to the present time there has been a retrograde movement, which has weakened and crippled the cause of higher education, and absolutely taken away from this great institution the resources and donations vouchsafed to it by the earliest acts of those who founded it and who considered its establishment and success indispensable to the proper and symmetrical growth of our whole educational system. This downward and degrading tendency has seemed to gather strength in an increasing ratio with each successive administration, until it reached its lowest and most contemptible exhibition in the illiberal and hostile attitude of the Twentieth Legislature, and the supine lethargy and apparently studied apathy of those who could and should have rendered valuable aid to so worthy an enterprise.

Contrast for a moment the temper and disposition of the present era in our State's history with the spirit which animated the souls and illumined the deeds of the founders of Texan liberty. That little handful of pioneers had scarcely conceived the idea of emancipation from the tyrannous yoke of an unfriendly government, and were yet struggling to establish an existence among the free people of the earth, when they proclaimed their devotion to the cause of higher education as the surest and only guarantee of a permanent and progressive system of popular self-government. As soon as their heroic effort was crowned with success, and the child of their genius and valor had taken its place among the equal States of the great federation of man, they were careful to embody in the organic law of the new government a special provision for the practical and early realization of the purpose they had from the first cherished on the subject of education. The first session of the Congress of the Republic was marked by a liberal donation of land to the University, and, as above noticed, the very location of the capital itself was accompanied by a designation of a site for the buildings of the future institution, which no one then seemed to doubt was to be the peculiar pride of our perfected scheme of free public instruction. The provisions of the Constitution of the Republic were repeated in that of 1845, under which the State was admitted into the Union of American States, and the continued sympathy and support of public opinion were steadily exerted to further the speedy organization of the University upon a scale commensurate with the design of its founders and with the place it was intended to fill in the completed system of State education. This enlightened and liberal spirit found expression in the act of 1858, above referred to, and, but for the unhappy interval caused by the civil war, would no doubt have found practical accomplishment in the inauguration of an institution which would now rank among the first in the land. Not the least of the evils brought about by the strife between the sections, seems to have been the lessened interest in the University wrought by the changed condition of public sentiment in Texas. Before the war, Texas was scarcely more than a frontier community, with less than one-fifth its present population, without railroads or any of the other means of industrial wealth now conspicuously developing throughout her borders. And yet no line of her statutes, and no single utterance of the men who then voiced the will of the people, were ever framed or promulgated in hostility to the University and its proper support and endowment. It remained for the generation that has come after the great civic revolution, to turn backward the current of our civilization, and to inaugurate a systematic spoliation of the funds and resources of the institution, and to decry and denounce its aims and objects with a senseless clamor and a contracted spirit of parsimonious enmity that have reduced it to a position of dependency so low that among the prevalent demagogues of the age there are "none so poor as to do it reverence."

The act of 1858 gave to the University one hundred thousand dollars in United States bonds in the State Treasury at that time; the fifty (50) leagues of land formerly appropriated under the act of 1839, and one out of every ten sections of land surveyed for State purposes, or under acts, general or special, granting lands to railroad companies. It is estimated that by the terms of this act, had they been complied with and their results not defeated by the fraudulent maneuvers of those who have from time to exercised the functions of legislators, the University would to-day have in its own right at least five millions of acres of land, worth on an average of five dollars per acre, making a grand total of twenty-five millions of dollars endowment. But what has become of this princely estate? When and where and by whom has this sacred trust been administered, or maladministered, so that there remains to the youths of Texas to-day only the worthless fragment of the legacy bequeathed to them by the fathers of their State? Until the act of 1881, under which the present Regency was organized and the University for the first time actually set in motion, the institution existed only in the patriotic provisions for its establishment embodied in the organic law of the Republic, and in the prophetic liberality and wisdom of those early statesmen, which have been honored only in their breach by the degenerate legislation of their unworthy successors. The University, having no tangible existence or legal representatives to protect its funds or husband its resources, was mercilessly plundered by the State during the somewhat chaotic condition of affairs after the war. It had no claims at that time, beyond the regard which every Texan should have had for the great objects foreshadowed by the founders of his State, and these were weakened by the sterner trials and temptations of the recent ordeal through which the whole country had passed.

At the time of the adoption of the Constitution of 1866, at the very lowest computation the number of acres then due and belonging to the University, under the act of 1858, was about one million six hundred thousand acres. That land is now worth from five to ten dollars per acre with an increasing value which is inestimable. All that land, by the Constitutions of 1866, 1869 and 1876, was absolutely taken away from the University and given to private corporations and to the public school fund. Thus, at one sweep, at least ten million dollars worth of land was taken from the University. The constitutional convention of 1875, in order to salve the wound and in fancied reparation of the pilfered property of the University, set apart one million acres of land which is not now worth one-half what the same amount would have been worth at the time it was taken from the University. The Legislature of 1883 gave an additional one million acres, but the land was undesignated, unsurveyed, and in fact is only of nominal value. In short and in plain language, the University has been robbed of its legitimate and valuable possessions and paid back "in chips and whetstones." Its funds and landed endowment, instead of being treated as a public trust, which in law and morals they have ever been, have been filched from and misappropriated with a shameless disregard of right and reason that, had it been exercised in reference to the administration of any private trust, would have been punished as a crime by the laws of the State. This conduct toward the sacred cause of higher education was in times past, no doubt, due in a large degree to a reckless want of attention to the legal and moral rights which, by virtue of its original conception and the provisions made for it by the fathers of the Republic and the State, the University was entitled to expect at the hands of the men who assumed to represent the people in the legislative halls of the State. Much can be excused on the score of ignorance and want of information on public questions, but when, as in the Legislature recently adjourned, a typical and controlling representative of the degenerate spirit of the age loudly and unblushingly proclaims that the University, being a creature of the State, has no rights that the State is bound to respect, and that its funds may be taken and the institution itself even abolished, then it is time that things should be called by their right names, and that those who are by law made the guardians of the interests of this great public enterprize should express themselves in no uncertain terms of indignant protest.

Thus repeatedly curtailed of its lawful income, the present resources of the University may be stated as follows:

State bonds, $523,511.63, of which the interest alone is available, being annually the sum of $31,949.38. Of these bonds, $186,000 worth will fall due in 1891, and will have to be reinvested at a lower rate of interest, which will probably reduce the income in the way of interest on these bonds to $26,479.38 per annum, instead of the above amount.

Land notes held in trust for the University by the State, $106,-810, on which the interest alone is available, amounting annually to $9,238.20. This, however, is an uncertain item of income, because these notes are being constantly and rapidly paid off, and in a few years the entire amount will be discharged, and will have to be reinvested at four per cent. interest per annum, which will reduce this source of revenue to $4,272.40 a year.

There is a cash item of $14.139.14, now to the credit of the University, which, being reinvested in four per cent. State or United States bonds, would yield an annual return of only $565.58, which, being added to the amount soon to be realized from above mentioned land notes, would leave in the near future a total yearly income of $4,837.98 from land notes and cash, instead of $9,238.20, as at present.

The two millions of acres appropriated by the Constitution of 1876 and the act of 1883, and which are vastly inferior in quality to the lands previously taken away, now constitute the whole landed property of the University, and, owing to defective legislation and the indifferent spirit exhibited by the State authorities, to whom the Regents have so often and earnestly appealed, are now absolutely unproductive as a source of revenue. All these facts were fully elaborated in the report of the Regents to the last Legislature, and it was asked that these lands be placed within the control of the Regency, who are best fitted to manage this estate in the interest of the institution, which is by law intrusted to their hands. It is a fact not open to controversy, that the University lands can never be profitably leased or disposed of if left under the control of the same authority that has the management of the public school lands. The two classes of lands are essentially different, and the policies regulating their management must be widely at variance. The University lands are in large bodies, mostly pastoral, situated within a limited territory, and without surface water; the school lands are in alternate sections, scattered over the entire State, largely agricultural and reasonably well watered. The same policy in reference to both is radically wrong and productive of great injustice to the University. Hence the request of the Regents that the lands belonging to the University be placed in the hands of the Board of Regents. Like every other appeal made to the last Legislature, upon the score of intelligent public policy and having for its object only the welfare of the State at large, this request was ignored, and even subjected to invidious criticism, characteristic of a body which is believed to have been phenomenal in the history of civilized legislation.

Again, it had been discovered that a large part of the one million acres granted by the act of 1883 was almost utterly worthless, and the Commissioner of the General Land Office very properly and generously made an exchange of this land for other land in El Paso county. The Twentieth legislature was asked to ratify this exchange, whose justice and propriety no one questioned; but, with a consistent contempt for enlightened public sentiment, the request was incontinently refused.

The present available income of the University is thus seen to be, from all sources, including the matriculation fees of students, $47,552.54. At the end of five years, owing to the necessity for reinvestment, above explained, the income from interest on the permanent fund will drop to $34,842.32 per annum. This amount is scarcely enough to maintain the institution in its present condition, much less to inaugurate other departments, or to properly extend and utilize those already in operation. Contrast this pitiful amount with the annual resources of other institutions with which, if we are to claim the name of a first-class university at all, we must necessarily refer for a comparative estimate of the funds required to properly conduct such establishments. The University of Virginia has an annual income of $90,000; the University of California, $103,000; the University of Michigan, $148,000, and Harvard College, $631,987.33.

And these are old institutions, fully equipped with libraries, buildings, apparatus, etc., and all the belongings which attach to well-established and prosperous colleges; and are, moreover, from year to year liberally assisted by private donations and public appropriations. The State Constitution of Texas prohibits any appropriations to the University from the general revenue of the State, and the only hope of income to the institution is from the utilization of its lands.

Out of the meager income thus left to the University, the Agricultural and Mechanical College annually receives, by act of the Legislature, $5000, which, with the decreasing amount of the annual revenues above explained, will reduce the University funds below the actual necessities of the institution, even in its present incomplete condition, and absolutely preclude the inauguration of its other departments and the proper maintenance of those already established.

Now, in reference to the A. and M. College, a few words will suffice. It is not believed that that institution should ever have been a part of the University of Texas. It constituted no part of the original conception of the men who provided for the endowment of a State University for higher education. It is an interjected experiment, dictated by the desire of the Federal government to interfere with the educational systems of the States, and was inaugurated at a time when the autonomy of the Southern States was in a situation of comparative paralysis. In an evil hour the prevailing demagoguism of the age incorporated it into our educational system, in the Constitution of 1876, and since that time it has been a fruitful theme of discussion for the ignorant, self-constituted champions of "farmers' rights," and a constant disturbing factor in the proper management of the University. Claiming to be a branch, it has pursued a policy of persistent hostility to the parent institution, and has arrogated to itself an undue importance not warranted by anything in its history or utility. Vaunting itself as the peculiar representative of the agricultural and mechanical class, its catalogues show that, during its whole existence, its students have come mainly from the towns and cities, and that only three out of all its graduates have ever resorted to farming or mechanical labor as a means of livelihood. Not satisfied to bear the burdens of poverty and straitened resources in common with other departments of the University, it clamors for a perfected organization and equipment far beyond what its own importance justifies or the financial condition of the institution warrants. Pandering to a false, but unfortunately prevalent sentiment, it has succeeded in acquiring a permanent property and an annual income at the expense of the rest of the University, out of all proportion to its due and proper place in the educational economy of the State. It has seven professors; the University has but ten. It has a students' dormitory, and wants another; the University has none. It wants a hospital and infirmary; the University, though equally in need of such a provision for the health and comfort of its students, has never felt justified in asking for it, in view of the limited resources at its command. And to crown the height of its extravagant claims, the last report of its Directory coolly asks the State to provide an appropriation to pay the students to labor in the fields and shops, which it is presumed were intended to teach the methods of manual skill and labor, but which the college authorities very ingenuously admit are comparatively unused, because of the aversion of the average student to work. Just think of it. The State is to furnish an institution to teach agricultural and mechanical pursuits, and then *pay* the boys to work at the tasks necessary and allotted to them under the system of instruction which they are supposed to be pursuing at the expense of the State. This very anomalous request had its origin, no doubt, in the fact that the students of the Bryan college are mostly city youths, and not the sturdy sons of country yeomen, who it is believed will never ask the State to pay them to perform the labor incident to a free tuition in their chosen devotion to the honest toil which underlies all civilization.

The A. and M. College, according to the last report of its Board of Directors, has now a permanent investment, in the shape of college buildings, apparatus, etc., of nearly $250,000. The main

University, at Austin, has expended only $59,000 for an uncompleted building, and its other investments will not raise the total amount spent to $100,000. The Bryan college has, from all sources, including Federal appropriations and the amount received from the University fund, an annual income between $69,000 and $70,000. The University proper has an income, after deducting the $5000 per year apppropriated to Bryan under the act of the last Legislature, of only $42,000, including the matriculation fees ot students, and not making allowance for the steadily decreasing revenues of the institution, resulting from the payment of land notes as before explained. The University, after being deliberately robbed of millions of acres of land which should justly have belonged to it under the act of 1858, has been paid off with two millions of acres, which are comparatively worthless, owing to the failure to place them under the control of the Regents, who are certainly as competent to manage them as any of the State authorities in whose hands, by existing law, they are exclusively vested. It is believed that, by reason of this omission, the institution, for the last three years, has lost annually at least $80,000, which might have been realized from the lease of these lands, and which, by this time, would have enabled us to establish a medical department. The last Legislature was earnestly besought to place this land in the hands of the Regents, but the request was refused, and the income from this source is so uncertain as not to be considered among the available resources of the institution.

The actual yearly income, then, of the University, is, in round numbers, $47,000 (including what goes to Bryan), and the positive expenditures of the departments, as now in operation at Austin, require every cent of this amount. The act of the Twentieth Legislature, appropriating $5000 per annum to the A. and M. College, will necessitate a reduction to that extent in the expenses of the University for the next two years. How this can be done without materially crippling the departments now in operation is a matter of serious concern to the Regents. In an elaborate and carefully prepared report, we went before the last Legislature and exhibited our condition in detail, and requested such legislation as would meet our wants. We asked that the University lands be placed in our hands, so that a revenue could be derived therefrom commensurate with their value, and which had heretofore been lost by the impolitic action of the State Land Board. We fully explained the exchange of the lands in El Paso county made by the Land Commissioner, and asked that his action be ratified. We set forth in full the facts pertaining to the $87,000 due and justly owing to the University from the State for money borrowed from its funds years ago, and whose validity has never been questioned, but repeatedly recognized and declared by the financial authorities of the State.

The inadequate accommodations of the present incomplete University edifice were made plain, and the necessity for new chairs of instruction and increased facilities in those already established was thoroughly discussed and demonstrated. It was shown that the University was living up to its very income, and that not a dollar could be diverted from it without serious embarrassment to its growth and efficiency.

Without exception, every single appeal made to the supposed representatives of the people was ignored; and, instead of acceding to our request for what was morally and legally our own, or even leaving us alone, the adjournment of the Twentieth Legislature found that "our last estate was worse than the first," and that the annual income of the institution is reduced to $5000 less than its actual expenses.

And this, too, when so important a branch as the Medical Department is yet unprovided for and clamoring for recognition.

And now, gentlemen of the medical profession, let me say what in this address it was my main purpose to say. Your interests are inseparable from the interests of the University in its full and symmetrical development. I am not insensible to the feeling which has obtained in certain quarters that the Regency has been unfriendly to the inauguration of the Medical Department. Nothing could be more unjust or erroneous than such an idea. In all that is said in this article it should be understood that nothing is meant unfriendly to the present location of the Medical Department, but is based on principle alone. It is true that we have believed that it was a fatal mistake ever to have separated the departments of the University, so that the prestige of the institution as a unit has been weakened and the antagonistic claims of particular localities have interfered to divide the house against itself, and well nigh wrecked the prosperity of the whole. If anything were needed to confirm our opinion in this regard, the experience we have undergone with the recent Legislature is enough. It was an open secret that Bryan and Galveston had entered into an alliance, offensive and defensive, against the main branch of the University at Austin. The debt of $87,000, due by the State to the University, was to be paid with an appropriation of $50,000, which should go to build the Medical Department at Galveston ; Bryan was to have her one-fifth of the University funds, Galveston would get a valuable building and permanent investment, which whould insure her in the location of the medical school for all time to come, and the University proper might look out for itself. With this ingeniously contrived scheme the Regents had nothing to do, but watched its concoction and denouement with a complacency born of repeated disaster. Like so many other coalitions between essentially different interests, the combination failed of achievement by the disagreement of the parties to it themselves, and Bryan got her usual quota from the University fund, while Galveston saw her hopes of a medical school fade into transparency—all of which goes to prove that in the proper development of this great institution, neither Austin, nor Bryan, nor Galveston should be considered. What is wanted is a State University of the first class, fully organized and and equipped in all its branches, and furnishing the standard of liberal learning and professional attainments for the whole State. Certainly the medical men of Texas have a right to expect and to demand a medical school representative of the professional aspirations of their extensive membership, and in keeping with the broadest culture of their science. Nothing less than this will satisfy them, and nothing less than this should be attempted. It is seriously doubted whether the State is under any obligation to furnish free professional education, either in law or medicine ; but if she does, she should furnish only the highest and best. The State cannot surely be expected to place her *imprimatur* upon quack doctors or pettifogging lawyers.

With this statement of the resources of the University, it is readily apparent that we cannot now successfully inaugurate the Medical Department. The Regents have been at some pains to ascertain what it would reasonably cost to properly conduct the medical school. The result of their investigations are contained in their last report to the Legislature, and are that it would require, exclusive of grounds, but including buildings, apparatus, etc., an original outlay of $140,000, and an annual expenditure of $40,000 for salaries of professors and other necessary expenses. It has been shown that the University, as at present situated, has no such sum at its disposal, but on the contrary, for the next two years will have to curtail its actual expenses $5,000 below what they now are. What, then, is the prospect and what the duty of the medical profession? Simply this: You must exert your influence to so mould public opinion that the University will be paid what is due it as an honest debt, and receive at the hands of future Legislatures such legislation as will enable it to properly utilize the mutilated fragment of its endowment left to it after the repeated spoliation practiced upon its estate as originally bequeathed to it by the fathers of Texas.

Had the requests of the Regents, preferred in their report to the Twentieth Legislature, been heeded, there might have been a chance to establish the Medical Department. But that appeal was ignominiously ignored, and those who presumably represented the local interest of that department apparently entered into a game of bargain and intrigue that materially crippled the entire institution, and accomplished nothing for the medical profession of Texas. There is but one course open to the physicians of this State. We need a medical school to educate the hundreds of young men who yearly go elsewhere for their professional education; we need it as a nucleus for scientific enterprise and research in our chosen calling; we need it as a measure of professional culture and a standard of professional attainment; we are entitled to it under the beneficent endowments of those who conceived the establishment of a State University, and we could have it to-day should the designs of those early patriots be fulfilled and the honest claims of the institution be recognized by those who now exercise the prerogatives of government.

The apathy of public sentiment in regard to the University is something appalling. For six years the Regents have labored in and out of season to properly administer the sacred trust committed to their keeping. Discouragement and defeat have led many to resign, and at times it has seemed that nothing was left us but to abandon the enterprise. Our repeated appeals to executive and legislative aid have been repeatedly disregarded, and our motives have been subjected to hostile criticism and misconstruction, such as should only be visited upon alien representatives of an unfriendly cause. Before the Legislatures of the State we have occupied the attitude of a foreign corporation, and in the secret cabals of local interests we have been treated as legitimate prey for whatever pillage chicanery could conceive or conspiracy consummate.

I appeal now to the medical profession of Texas. The proper establishment and successful operation of the departnent in which you are peculiarly interested, depend upon the concession of all that the Regents have petitioned for. Every dollar diverted from the University fund to branches already unduly favored, is so much taken from your interest in the heritage vouchsafed to you by wisdom of those who founded the institution and framed our earlier legislation for its best and highest development. The University is one and inseparable, and its authorities ask nothing but what is in law and morals its own, and they demand for it only an enlightened public recognition of its importance as a controlling factor in the State's civilization.

